# Fruit Extract Derived from a Mixture of Noni, Pineapple and Mango Capable of Coagulating Milk and Producing Curd with Antidiabetic Activities

**DOI:** 10.17113/ftb.60.03.22.7456

**Published:** 2022-09

**Authors:** Jaya Vejayan, Rupbansraaj Bathmanathan, Sharifah Aminah Tuan Said, Srikumar Chakravarthi, Halijah Ibrahim

**Affiliations:** 1 Faculty of Industrial Sciences & Technology, Universiti Malaysia Pahang, Lebuhraya Tun Razak, 26300, Gambang, Kuantan, Pahang Darul Makmur, Malaysia; 2 Faculty of Medicine, Biomedical Sciences and Nursing, MAHSA University, Jalan SP2, Bandar Saujana Putra, 42610 Jenjarom, Selangor, Malaysia; 3 Institute of Biological Sciences, University of Malaya, 50603 Kuala Lumpur, Malaysia; # Present address: SEGi University & Colleges, No. 9, Jalan Teknologi, Taman Sains Selangor, Kota Damansara, PJU 5, 47810 Petaling Jaya, Selangor, Malaysia

**Keywords:** milk coagulation, antidiabetic properties of curd, streptozotocin-induced diabetes, *Morinda citrifolia*, *Mangifera indica*, *Ananas comosus*

## Abstract

**Research background:**

*Morinda citrifolia* L. (noni), *Ananas comosus* L. cv. Sarawak (pineapple) and *Mangifera indica* L. cv. Apple (mango) represent fruits capable of coagulating milk and forming a curd. Plant-derived milk coagulants have antidiabetic phytochemicals that enrich the curd. Hence this work evaluates the dual benefits of the fruits in coagulating milk and the antidiabetic activities found in the obtained curd.

**Experimental approach:**

The three fruits were mixed to form a supercoagulant (a milk coagulant mixture of the extracts at a ratio of 1:1:1), and the milk coagulation time was measured. The milk was coagulated by the supercoagulant, and thus fortified curd was tested for its ability to inhibit α-glucosidase and α-amylase activities. Then, the fortified curd was fed daily to streptozotocin-induced diabetic rats and their biochemical markers such as blood glucose level, aspartate aminotransferase, alanine transaminase, *etc.* as well as histopathology of their liver and kidney tissues were compared with the untreated diabetic rats and normal rats.

**Results and conclusion:**

The supercoagulant had a milk coagulation time of (28±3) s at a 50 mg/mL concentration. Its fortified curd inhibited α-glucosidase and α-amylase activities, with IC_50_ values of (4.04±0.03) and (3.42±0.02) mg/mL, respectively. The average mass of the streptozotocin-induced diabetic rats fed daily with curd formed by the supercoagulant was (201±10) g on day 20 compared to diabetic control rats with (149±16) g. The blood glucose concentration for rats treated with the supercoagulant after fasting was (15±1) mmol/L compared to the diabetic control rats ((26±2) mmol/L). Blood tests on the treated rats showed aspartate aminotransferase, alanine transaminase, γ-glutamyl transferase and alkaline phosphatase (liver function tests) amounts of (214±78), (91±13), 3 and (510±138) U/L, respectively, while the total protein and renal function tests showed the concentrations of albumin, globulin, urea and creatinine of (37±2) g/L, (30±2) g/L, (11±1) mmol/L and (42±3) µmol/L, respectively. These concentrations were found to be similar to those of the normal rats on day 20. Furthermore, a histopathological study performed on the liver and kidney of the rats found no apparent damage.

**Novelty and scientific contribution:**

This supercoagulant derived from a mixture of fruits is able to coagulate milk rapidly, and its curd is fortified with safe antidiabetic agents. The supercoagulant is potentially useful in producing functional dairy food to prevent diabetes or as a supplement for diabetics to control their blood sugar. Such products are capable of replacing dairy products derived from animal enzymes and provide consumers with additional functional dairy products.

## INTRODUCTION

Milk and dairy products are known for their health benefits due to the biologically active components they contain, including bioactive peptides, organic acids, vitamins and others. Curd is a type of dairy product formed from the curdling of milk through coagulation. The most common protein found in mammalian milk is casein. The clotting of milk can be described as the clumping of casein due to the distorting effects of the coagulant that eventually cause the formation of gel-like structures capable of absorbing some water as well as trapping fat globules (*1*). In general, milk coagulation can be achieved by the addition of enzymes, acid treatment or heat-acid treatment. In cheese manufacturing, enzymatic coagulation of milk by chymosin (rennet) is widely used, an enzyme typically isolated from the stomach of young ruminant animals, a process that may be considered unethical. Other sources of milk coagulant are extracted from animal sources, including adult cows and pigs, which has come into conflict with religious beliefs. Moulds, mostly genetically engineered, that have the ability to produce proteolytic enzymes are used to produce microbial rennet as an alternative to animal-sourced coagulants. However, these products have raised serious concerns, as genetically modified foods have been claimed to cause life-threatening allergic reactions and they have been banned in France, Germany and the Netherlands (*2*).

The globally increasing demand for dairy products such as cheese, yogurt and others has led to research seeking an alternative source of coagulant to form curd, and one such source is plants. Many plant coagulants have been identified to have milk-coagulating capabilities and therefore they can be used as an alternative to commercial coagulants (*3*). However, plant-derived coagulants come with problems such as the longer time needed for a complete coagulation process than rennet, a potential bitter flavour and an inconsistent texture of the curd (*2*). These challenges can potentially be overcome by combining a number of plants known for their ability to coagulate milk. Therefore, a ‘supercoagulant’ was made of plant extracts to enhance and improve the coagulation of milk. Supercoagulant is a simple term given within this report to describe a combination of three plant extracts made up of noni (*Morinda citrifolia*), pineapple (*Ananas comosus*) and mango (*Mangifera indica*). The supercoagulant, apart from coagulating the milk, is expected to enrich the formed curd with secondary metabolites or phytochemicals with medicinal properties.

Secondary metabolites play important roles in plants, including defence or preventive mechanisms against infections and diseases. Once plants are consumed by humans and animals, phytochemicals are useful and they have many biological capabilities. One such potential is the prevention of diabetes, a disease that is becoming a major public health concern. Diabetes, especially the type 2, is of concern to people consuming food rich in sweeteners. The International Diabetes Federation (IDF) stated that the prevalence of diabetes among Malaysian adults over 18 years of age was 16.9%, with 3.49 million reported cases of diabetes in 2017 (*4*). α-Glucosidase and α-amylase are the key enzymes involved in the hydrolysis of carbohydrates to glucose and other monosaccharides. Due to the absence or ineffectiveness of insulin found in diabetic patients, the hydrolysis of carbohydrates can cause an accumulation of glucose, leading to hyperglycaemia (*5*). To counter this, these enzymes need to be inhibited, and it has been found that the phytochemicals made by plants are capable of doing this (*6*-*8*). Many studies have been done on the antidiabetic properties of plants, but none have investigated the curd that is formed by coagulation of milk with plants. Hence, this study focuses on milk coagulation by the supercoagulant to form curd and its antidiabetic properties.

## MATERIALS AND METHODS

### Sample

Noni (*Morinda citrifolia* L.), pineapple (*Ananas comosus* L. cv. Sarawak) and mango (*Mangifera indica* L. cv. Apple) were obtained from various cultivators in the region of Kuantan, Malaysia, and their voucher specimens were deposited at the Institute of Biological Sciences, University of Malaya, Malaysia with voucher numbers HI1445, HI1446 and HI1447, respectively. The seeds (seed coat removed) of mango and the fruits of noni and pineapple were washed thoroughly to remove unwanted contaminants and then cut into pieces. The mango seeds were dried in an oven (Universal oven UN55; Memmert GmbH, Schwabach, Germany) with convection at 50 °C until dry and brittle enough to be pulverized into powder. Noni and pineapple fruits were used fresh to avoid spoilage by fungi under drying.

### Preparation of extracts

A total of 10% (*m*/*V*) powdered mango seeds were mixed in water for aqueous extraction. The mixture was mixed at a constant speed in a magnetic stirrer (Nuova II Stir Plate; Thermolyne Corporation, Dubuque, IA, USA) for 5 h before removing large particles with a coarse muslin cloth filter followed by fine filtration with Whatman grade 1 filter paper (Sigma-Aldrich, Merck, St. Louis, MO, USA). The supernatant was freeze-dried (Labconco™ FreeZone™ Console freeze dryer; Thermo Fisher Scientific, Kansas City, MO, USA) and kept until further use. Each sample of noni and pineapple at 10% (*m*/*V*) in water was squeezed in a perforated stainless-steel filter and consequently fine filtered through Whatman grade 1 filter paper (Sigma-Aldrich, Merck) to obtain their sap before freeze-drying.

### Supercoagulant formation

Noni, pineapple and mango extracts were combined at a ratio of 1:1:1, *i.e*. 10 g each was homogenized and dissolved completely in 500 mL of deionized distilled water. This mixture was then freeze-dried to obtain the supercoagulant powder, which was used throughout this study. Type II rennet, with chymosin as the main constituent, sourced from *Rhizomucor miehei* (Sigma-Aldrich, Merck) was used as the coagulant control for comparison.

### Determining the milk coagulation time

The time was recorded after confirming three parameters: the change in viscosity and colour, and the appearance of white spots in a drop of milk supplemented with coagulant, which were verified under a light microscope (Eclipse E100 fitted with a digital imager DinoEye eyepiece camera software; Nikon, New York, NY, USA) at intervals of every 5 s. The tested milk mixture included 1 mL of 10% (*m*/*V*) skimmed milk (BD Difco, Franklin Lakes, NJ, USA) with 0.5 mL of either 5% (*m*/*V*) supercoagulant or 1% (*m*/*V*) rennet and 10 mM CaCl_2_ (Sigma-Aldrich, Merck) at pH=6.5, 35 °C. Experiments were performed in quadruplicate. The pH=6.5 was used as it is the natural pH of milk, while CaCl_2_ functions as a cofactor for milk coagulation by promoting the aggregation of casein micelles (*9*). The concentration of supercoagulant was 5% (*m*/*V*) and of rennet 1% (*m*/*V*) to achieve a standard coagulation initiation time of about 30 s for both coagulants as it is ideal for laboratory conditions for the purpose of analysis. A choice of too rapid coagulation initiation time was found to complicate the analysis and comparison between the coagulants.

### Determining the visible changes and measuring the calcium content of the coagulated milk

The observations were performed on milk coagulated with the supercoagulant, milk coagulated with rennet and milk alone (control). In each test, 1 mL of 10% (*m*/*V*) skimmed milk (BD Difco) was treated with 0.5 mL of either 5% (*m*/*V*) supercoagulant or 1% (*m*/*V*) rennet or milk alone under conditions of pH=6.5, 35 °C and 10 mM CaCl_2_ (Sigma-Aldrich, Merck). The samples were centrifuged at 10 062×*g* for 60 s using a microcentrifuge (Z 216 MK; Hermle, Wehingen, Germany) to separate the curd and whey for comparison with each other. Next, in separate tests, structural changes were observed using a standard light microscope (Eclipse E100 fitted with a digital imager DinoEye Eyepiece camera software; Nikon) at 100× magnification. Finally, the structural changes were also viewed at a higher resolution using a scanning electron microscope (Fei Quanta 50; Thermo Fisher Scientific).

For determining the amount of calcium, all parameters were similar to those for the evaluation of the visible changes except without the addition of CaCl_2,_ as this study investigated the contribution of the *in situ* calcium present in milk. After being left overnight for complete coagulation to take place, the curd together with whey and the milk alone were freeze-dried before measuring the calcium content by an inductively coupled plasma-optical emission spectroscopy (ICP-OES; Optima 7300 DV; Perkin Elmer Inc., Shelton, CT, USA).

### Determination of antidiabetic activity of curd fortified with the supercoagulant

**Fig. S1:**
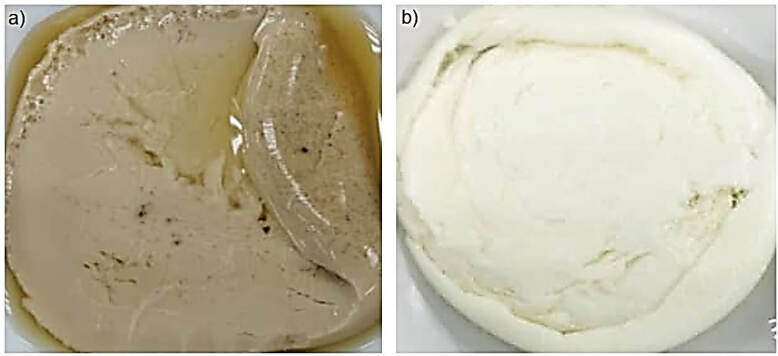
Photographs of: a) supercoagulant-fortified curd, and b) renneted curd obtained after coagulation of milk and removal of whey

The supercoagulant-fortified curd (Fig. S1a) was prepared by allowing 5% (*m*/*V*) supercoagulant to react overnight with 10% (*m*/*V*) skimmed milk (BD Difco) at pH=6.5, 35 °C and 10 mM CaCl_2_. The mixture was centrifuged (Z 216 MK; Hermle) at 10 062×*g* for 2 min to remove the whey; only the curd was freeze-dried and used for further study. Similarly, the prepared curd formed with 1% (*m*/*V*) rennet (Fig. S1b) was used as a control.

### α-Glucosidase inhibitory assay

This assay was a modification of a previous method (*10*). Briefly, an equal volume of various sample aqueous solutions was mixed with 1 U/mL enzyme (α-glucosidase isolated from *Saccharomyces cerevisiae*, Sigma-Aldrich, Merck) in potassium phosphate buffer, pH=6.9, and incubated for 30 min at 37 °C. This was followed by an additional 15 min of incubation after adding 10 mM *p*-nitrophenol-α-glucopyranoside (Sigma-Aldrich, Merck). The reactions were stopped by adding 100 mM Na_2_CO_3_ (Merck, Darmstadt, Germany) to the mixture. The tested samples included supercoagulant-fortified curd, renneted curd and acarbose (100 mg of Glucobay tablet, Bayer Corp., Whippany, NJ, USA), and a control without any sample. All of the tests were conducted in multiples of five for the calculation of standard errors. The absorbance was measured at 405 nm using microplate reader (Infinite 200 PRO equipped with Magellan software; Tecan, Männedorf, Switzerland) and the inhibitory activity (in %) was calculated with the following equation:



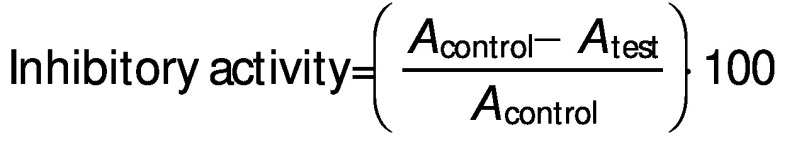



where *A* is the absorbance at the specified wavelength.

A graph of enzyme inhibitory activity (in %) *versus* the concentration of the sample was used in estimating the IC_50_ value.

### α-Amylase inhibitory activity assay

The assay was a modification of a previous method (*11*). Briefly, 250 µL samples in increasing concentrations were mixed with 50 µL of 2 U/mL enzyme (α-amylase isolated from pig, Sigma-Aldrich, Merck) in potassium phosphate buffer (Sigma-Aldrich, Merck), pH=6.9, and incubated for 30 min at 25 °C. Next, the cells were incubated for an additional 10 min once starch (1% *m*/*V*) (Sigma-Aldrich, Merck) was added as a substrate for the reaction. The reaction was terminated by adding 50 µL 3,5-dinitrosalicyclic acid (DNS, Sigma-Aldrich, Merck) at 85 °C and immediately cooling for 5 min to room temperature. The tested samples included supercoagulant-enriched curd, renneted curd and acarbose (100 mg of Glucobay tablet, Bayer Corp.), and a control without any sample. All tests were conducted in multiples of five for the calculations of standard errors. The absorbance was measured at 540 nm using microplate reader (Infinite 200 PRO equipped with Magellan software; Tecan), the inhibitory activity (in %) was calculated with Eq. 1, plotted *versus* the concentration of the sample and used for estimating the IC_50_ value.

### Determining the effects of the supercoagulant curd in rats induced with diabetes

Approval to conduct an *in vivo* study was obtained from the Animal Ethics Committee of Universiti Malaysia Pahang (Approval No. UMPIACUC/2020/02) for the use of 12 Sprague-Dawley male rats. The rats, weighing between 200 and 300 g, were allowed to acclimatize for two weeks to laboratory conditions. The experiment involved three groups of four rats in each group: normal control (NC), diabetic control (DC) and supercoagulant-enriched curd (SC). Diabetes conditions were induced by streptozotocin (Sigma-Aldrich, Merck) using a previously described method (*12*). Briefly, the rats in the DC and SC groups were injected IP (intraperitoneally) with 60 mg/kg streptozotocin dissolved in 50 mM sodium citrate buffer (Sigma-Aldrich, Merck) at pH=4.5. Throughout the day, the rats were given 10% (*m*/*V*) sucrose (Sigma-Aldrich, Merck) in water and standard food pellets (Gold Coin Feed Mills Sdn. Bhd., Kuala Lumpur, Malaysia) for the initiation of the hypoglycaemic effect. The next day, they were given water and food *ad libitum*. Their initial mass and blood glucose concentrations were measured before (day 0) and after (day 5) inducing diabetes, with the blood glucose concentration of the latter being greater than 22 mmol/L.

The experiment commenced by giving the NC and DC groups 2 mL of water alone, while SC group was dosed with 300 mg/kg supercoagulant-enriched curd daily using oral gavage for a total of 20 days. Consequently, the mass and blood glucose after fasting were measured (Accu-Check Performa Glucometer, Roche Diabetes Care, Mannheim, Germany) for each rat at intervals of every 5 days. On day 20, the rats were sacrificed humanely by overdose with a ketamine and xylazine mixture (Laboratory Animal Science Association of Malaysia (LASAM), Universiti Kebangsaan Malaysia (UKM), Malaysia). A total of 1.0 mL blood was drawn directly from the heart once the midline of the abdomen was cut open to reveal the abdomen cavity. Next, the liver and right kidney were dissected and preserved in 10% formalin (Merck). The blood was sent in vacutainers for biochemical parameter diagnostics at Gribbles Pathology Laboratory, Malaysia. The organs were treated with xylene, embedded in paraffin wax, microtome sliced into 5-micron sections, then stained with haematoxylin and eosin, and the prepared slides were carefully graded under a light microscope (Eclipse TS100; Nikon) by an expert pathologist as described previously (*13*).

### Statistical analysis

Data were analysed using IBM Statistical Product and Service Solutions (SPSS) Statistics v. 25 tools (*14*). All of the samples were assayed in multiples of five for the calculation of standard errors and data were expressed as mean value±standard error. The results were considered significant at p<0.05. An independent *t*-test was used to compare the data within the group, while one-way ANOVA was used to compare the data between groups, followed by Tukey’s *post hoc* test for multiple group comparison.

## RESULTS AND DISCUSSION

### Milk coagulation and fortification of the curd

The supercoagulant contains extracts from noni (*Morinda citrifolia* L.), pineapple (*Ananas comosus* L. cv. Sarawak) and mango (*Mangifera indica* L. cv. Apple) mixed in a ratio of 1:1:1. The ratio was selected as the protein and phytochemical contents within the fruits vary from batch to batch and therefore, an equal mixing of the three fruits is required so that they can contribute equally to the milk coagulating ability and biological activity of the supercoagulant. These fruits have the ability to coagulate milk due to the presence of milk-coagulating proteases. It was found that 5% (*m*/*V*) supercoagulant had a coagulation time of (28.3±2.9) s, while 1% (*m*/*V*) rennet had a coagulation time of (31.7±2.9) s (data not shown). Rennet (a commercial milk coagulant) was chosen to be used at a random amount five times lower for testing than supercoagulant, as the former is a purified protease, while the supercoagulant crude plant extracts are known to contain mostly non-protein constituents. Earlier studies have proven that milk-coagulating proteases are present in each of the fruits that make up the supercoagulant. Studies have shown that noni contains proteases that are able to coagulate milk, while bromelain, a protein commonly found in pineapple, has also been found to have milk-coagulating ability (*15*, *16*). Previous studies have also shown that mango has milk-coagulating capability due to the proteases found in it (*17*).

**Fig. 1:**
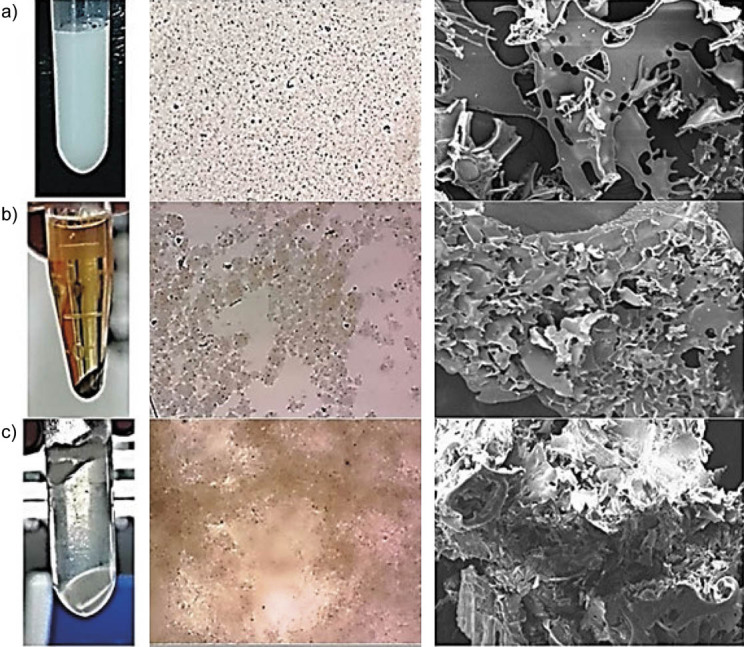
Observed changes in: a) pure milk, b) milk coagulated with supercoagulant, and c) milk coagulated with rennet. From left to right: after centrifugation at 10 062×*g* for 60 s, under a light microscope at 100× magnification, and SEM micrograph at 500× magnification, respectively

Fig. 1 shows the visible changes in pure milk, milk coagulated with supercoagulant and milk coagulated with rennet after centrifugation, light microscope analysis and scanning electron microscopy (SEM) analysis. Separation was not observed in pure milk (Fig. 1a), while clear separation of solid curd and liquid whey was observed in the milk supplemented with supercoagulant (Fig. 1b) and rennet (Fig. 1c) after centrifugation (*18*). During the microscopic analysis, the pure milk (Fig. 1a) shows a confluent structure without dark spots or aggregation of casein, while the milk samples coagulated with supercoagulant (Fig. 1b) and rennet (Fig. 1c) show obvious dark spots and colloidal structures of casein micelles. Results of SEM analysis in Fig. 1a show that the pure milk has a smooth surface with observable hair-like structures, which could be casein micelles with κ-casein (*19*). Spaced-out structures can be seen that show no aggregation of molecules and thus no coagulation. On the other hand, milk coagulated with supercoagulant (Fig. 1b) and rennet (Fig. 1c) display a rough surface without clear see-through spaces or translucent areas between the structures because the particles aggregated together due to coagulation. The dark aggregated spots shown during light microscopy and SEM analyses represent the aggregated casein micelle that would form the curd, while the clear spaces between them represent the whey (*20*). It can also be seen that rennet forms a denser curd than supercoagulant, as there are fewer spaces between the structures. This is because rennet is a purified protease and therefore it cleaves κ-casein at the Phe_105_–Met_106_ bond in the casein micelles more effectively than the supercoagulant, forming a more complex three-dimensional network of chains and clusters (*21*). A study also reported that low proteolytic activity forms a denser protein network, which is why the rennet-coagulated milk structure is denser (*22*).

**Fig. 2:**
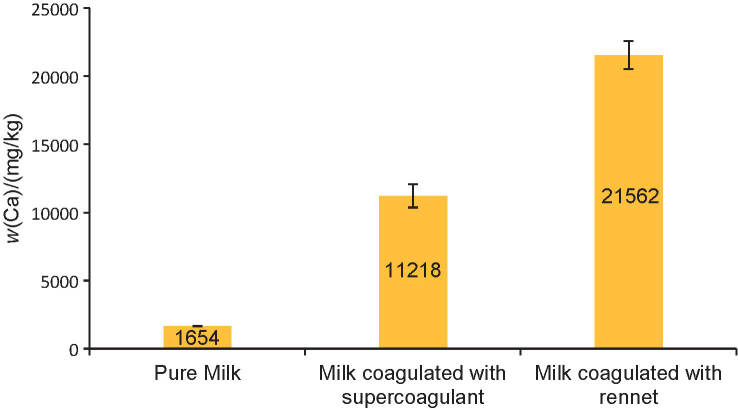
Determination of calcium with inductively coupled plasma-optical emission spectroscopy (ICP-OES). The experiment was performed in quadruplicate, and the values are expressed as the mean±standard error, where p<0.05

The casein micelles are made up of casein submicelles that contain colloidal calcium phosphate clusters and a ’hairy’ layer of protruding κ-casein, which gives the micelle steric and electrostatic stability (*23*). The function of calcium phosphate nanoclusters in milk is to prevent precipitation and calcification of the milk and to interlock the protein strands within the casein micelle (*24*). During cheese-making, a specific protease is added to the milk to cleave κ-casein on the surfaces of the casein micelles, which lowers the repulsive forces between them and causes aggregation (*23*). Theoretically, when κ-casein is cleaved, calcium phosphate nanoclusters are also disrupted and solubilize, which would lead to an increase in the total calcium content within the curd. Calcium is an essential element in the secondary stage of coagulation, where it helps with the aggregation of casein micelles (*25*). Fig. 2 shows the pure milk, the milk coagulated with supercoagulant and with rennet after the determination of calcium content using ICP-OES. The mass fraction of calcium in pure milk was 1654 mg/kg, and this value is similar to a previous study, which found that commercial skimmed milk in South Korea contains calcium 1184 mg/kg (*26*). Accordingly, a surge in calcium release was detected, and its mass fraction in the rennet-coagulated milk was almost twice as high as the calcium mass fraction of the supercoagulant-coagulated milk. This may be because rennet is a purified form of protease; therefore, the cleavage and coagulation it carries out is much more efficient and specific than that of the supercoagulant, which is in a crude form.

### In vitro antidiabetic activities of fortified curd

**Fig. 3:**
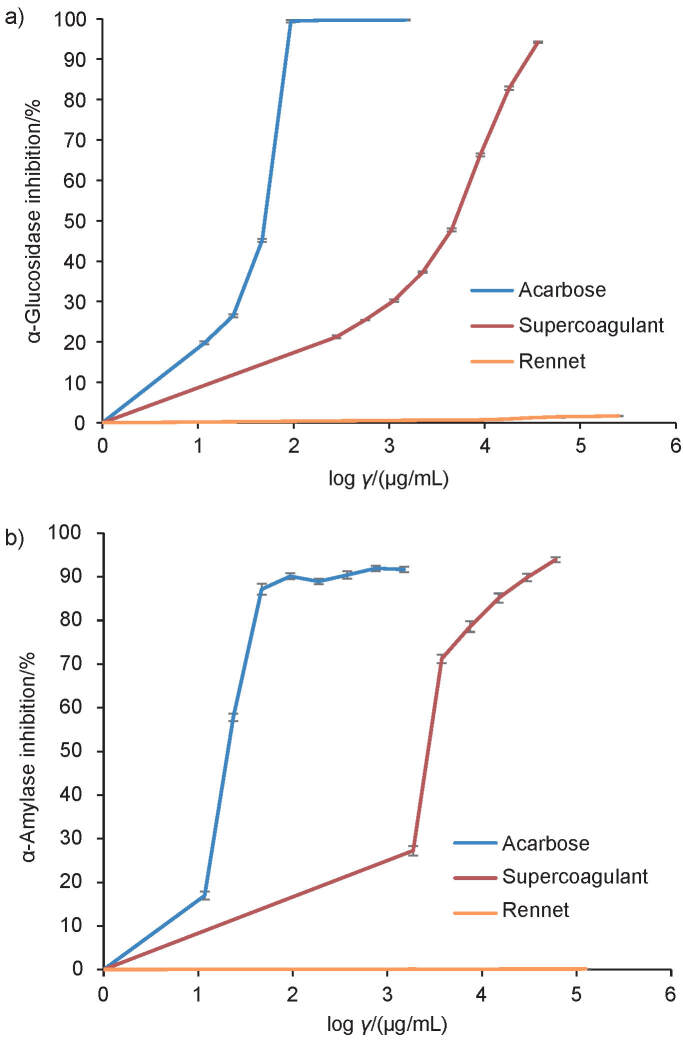
The inhibition of: a) α-glucosidase, and b) α-amylase with different log concentrations of the tested samples. The experiments were performed in quadruplicate, and the values are expressed as the mean±standard error, where p<0.05

The inhibition of two important polysaccharide-degrading enzymes, α-glucosidase and α-amylase, by the supercoagulant curd is shown in Fig. 3. It was compared with acarbose, a common drug used to treat type 2 diabetes mellitus. The IC_50_ values of acarbose and the supercoagulant-enriched curd for α-glucosidase were (0.05±0.02) and (4.04±0.03) mg/mL, respectively. The IC_50_ values for α-amylase were (0.03±0.02) and (3.42±0.02) mg/mL, respectively. The supercoagulant-enriched curd was found to have significant antidiabetic properties even though its IC_50_ value was comparably higher than that of acarbose. The supercoagulant-enriched curd had an IC_50_=4.04 mg/mL while the rennet curd shows no inhibitory activity. This is due to the presence of phytochemicals within the plant extracts that give the supercoagulant curd its antidiabetic properties. Mango seed extract and the noni fruit extract have been reported to possess excellent antidiabetic activity based on the studies conducted in tissue culture and animal models where they were found to inhibit the α-glucosidase and α-amylase enzymes, and reduce the blood sugar level (*27*).

For α-glucosidase inhibitory activity, mango seed extract and noni fruit extract have been found to have IC_50_ values of 0.34 and 0.2 mg/mL, respectively (*27*, *28*). Similarly, for α-amylase inhibitory activity, mango seed extract and noni fruit extract have been found to have IC_50_ values of 0.71 and 2.62 mg/mL, respectively (*27*-*29*). When compared with the literature, α-glucosidase and α-amylase inhibitory activities of the supercoagulant-enriched curd were not as strong as those of its individual fruits. The possible differences between the IC_50_ values may be due to several factors, one being the leaching out of the antidiabetic components into the discarded whey during curd production (*30*). Other factors include differences in ripeness, varieties of fruits, and growth conditions (*31*). Nevertheless, the supercoagulant-enriched curd was still able to provide significant antidiabetic activity as opposed to rennet curd, which does not have inhibitory potency.

### In vivo antidiabetic activities and safety evaluations of the fortified curd

**Fig. 4:**
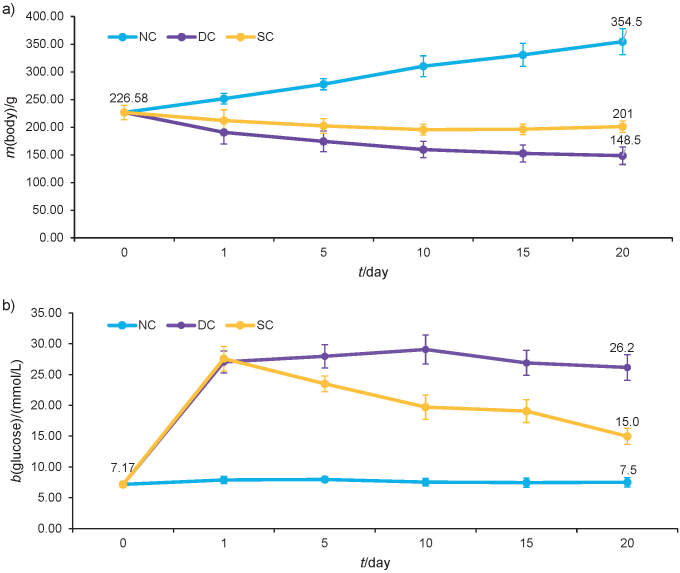
*In vivo* evaluations of: a) mean body mass of the rats, and b) mean fasting blood glucose concentration during 20 days. The experiments were performed in quadruplicate, and the values are expressed as the mean±standard error, where p<0.05

The supercoagulant-enriched curd was then subjected to *in vivo* testing of its antidiabetic properties in rats. Fig. 4 shows the mean body mass and glucometer measurements of the fasting blood glucose level in normal control (NC), diabetic control (DC) and supercoagulant-enriched curd (SC) rats. The parameters showed no changes on day 0 for NC or for both DC and SC groups prior to the diabetes induction of the rats (p>0.05). The mass of NC rats progressively increased, being the highest on day 20 with a mean body mass of (355±24) g. Growth can be defined as a progressive increase in body mass of an animal during a specific time period due to the accumulation of proteins and fat and bone formation in the animals, which is why there is a constant increase in body mass in the NC group (*32*). On day 20, the DC group of rats had a mean body mass of (149±16) g, an unfavourable reduction of 34% compared to the initial mean body mass of (227±13) g. The SC group of rats also showed a reduction of mean body mass, but only by approx. 11%. The mean body mass of diabetic rats was found to decrease over time as the pancreatic β-cells were damaged, causing lower insulin production. This prevents the breakdown of glucose in body cells; therefore, the body cannot use glucose as a source of energy but instead uses proteins and fats in the body, which leads to a loss of body mass (*33*). However, oral treatment of the rats with supercoagulant-enriched curd reduced the drastic mass loss observed in the untreated animals.

The fasting blood glucose concentrations increased in DC and SC groups on day 1 when diabetes was induced in the rats. The DC blood glucose concentration remained high and somewhat flat throughout the measurements until a final measurement of (26±2) mmol/L in comparison to SC, where it was reduced to (15±1) mmol/L; however, this value was still found to be almost twice higher than the value of NC, (8±1) mmol/L, on day 20. All of the differences in the measurements of DC and SC were found to be statistically significant compared to NC (p<0.05). The administration of streptozotocin destroys pancreatic β-cells and causes less insulin to be released into the body, which leads to the accumulation of glucose and the development of constant hyperglycaemia (*34*). The hydrolysis of carbohydrates to glucose and other monosaccharides in the body is caused by enzymes such as α-glucosidase and α-amylase. Due to the absence or ineffectiveness of insulin, the hydrolysis of carbohydrates causes an accumulation of glucose. Antidiabetic drugs such as acarbose are used to inhibit these enzymes so that the glucose level remains low in the body (*5*). Supercoagulant-enriched curd was found to have a similar effect to these drugs, as it was also found to lower the blood glucose concentration in the SC group rats. Studies have shown that certain plants have the ability to inhibit the α-glucosidase and α-amylase enzymes to lower blood glucose concentration, and similarly, the supercoagulant-enriched curd was found to have this ability during *in vitro* antidiabetic studies (*35*). The supercoagulant was able to reduce the increasing fasting blood glucose concentration, as evident in the diabetic rats after day 20.

Several biochemical diagnostic parameters were evaluated in the rat blood and are shown in Table 1. The measured amounts of aspartate aminotransferase, alanine transaminase, γ-glutamyl transferase and alkaline phosphatase were significantly the highest in the DC group (p<0.05). The amounts of aspartate aminotransferase, alanine transaminase and γ-glutamyl transferase were similar to those in SC rats, while alkaline phosphatase amounts were found to be low compared to those in untreated DC rats.

**Table 1:** Changes in blood biochemical parameters on day 20 of normal rats (NC), rats with streptozotocin-induced diabetes (DC) and rats fed curd with supercoagulants (SC)

Blood parameter	Unit	NC	DC	SC
Aspartate aminotransferase	U/L	221.5±86.9	926±143	214.0±78.8
Alanine transaminase	U/L	52.0±10.2	460.8±94.2	91.0±12.6
γ-glutamyl transferase	U/L	3.5±1.0	13.0±2.9	3.00±0.00
Alkaline phosphatase	U/L	180.5±40.7	944.5±37.8	509.8±38.3
Albumin	g/L	38.5±2.9	26.0±1.2	37.2±1.9
Globulin	g/L	29.5±4.6	25.25±2.1	30.2±2.2
Total protein	g/L	68.0±1.8	51.2±3.0	67.5±1.7
Urea	mmol/L	7.0±1.2	19.6±1.9	11.0±1.1
Creatinine	µmol/L	40.2±4.1	44.0±3.4	42.2±2.8

The values are expressed as the mean±standard error, where p<0.05

When liver tissues are damaged, additional enzymes are released into the bloodstream that increase the enzyme amount in the blood (*36*). The enzymes in the DC group were significantly higher than those in the other groups due to liver cell injury by streptozotocin in diabetic rats, which increased the release of liver enzymes into the blood (*37*). On the other hand, the SC group had enzyme amounts that were significantly lower than those of the DC group (p<0.05), which shows that even though the SC group was initially diabetic, treatment with the supercoagulant-enriched curd helped lower the diagnostic enzyme levels. The treatment was effective in lowering all of the enzyme amounts of the diabetic rats to almost normal levels except for alkaline phosphatase. The alkaline phosphatase amount of the SC group did not decrease as much as that of the other enzymes, but it still showed a significant reduction compared to the DC group.

The same trend was also observed for the tested protein concentrations, whereby the albumin, globulin and total protein concentrations were similar in NC and SC, but were significantly reduced in the DC group of rats (p<0.05). A total protein test was used to determine the amount of protein in the blood and to diagnose liver and kidney functions. If the total protein concentration is low, there may be a liver or kidney problem because the protein is not digested and absorbed properly by the body (*38*). The serum albumin concentration in the DC group was significantly lower than that in the NC group, possibly due to increased urinary excretion or low hepatic synthesis of albumin (*39*). However, this was not found in the SC group, as the albumin concentration was significantly higher (p<0.05) than that in the DC group and similar to that in the NC group. The DC group also showed lower concentrations of globulin than the NC and SC groups, but the difference was not significant (p>0.05). The total protein concentration is the measure of the total amount of albumin and globulin in the body, and the total protein concentration of the DC group was significantly lower than that of the NC and SC groups (p<0.05). This is because in diabetic rats, intense changes in protein metabolism and loss of nitrogen from organs take place, which causes a negative nitrogen balance (*40*). This was not seen in the SC group, as the protein concentration was found to be significantly higher due to the therapeutic effects of the supercoagulant-enriched curd.

For renal function diagnostics, urea and creatine were the highest in DC rats compared to NC and SC rats. A renal function test was used to determine the function and damage that occurred in the kidney due to diabetes in the rats. Urea and creatinine are waste products in the blood that are eliminated by the kidney. A high level of these waste products in the blood indicates damage to the kidney due to diabetes (*41*). The DC group had a significantly higher urea concentration than the NC group (p<0.05), which shows that the urea concentration in the blood was high due to significant renal damage. The rats in the SC group showed significantly lower urea concentrations (p<0.05) than those in the DC group. The DC group also showed the highest creatinine concentration compared to the other groups due to renal damage, which caused an accumulation of creatinine in blood. These concentrations of SC were comparable to those of the NC group, which shows that the treatment was effective in improving kidney function.

**Fig. 5:**
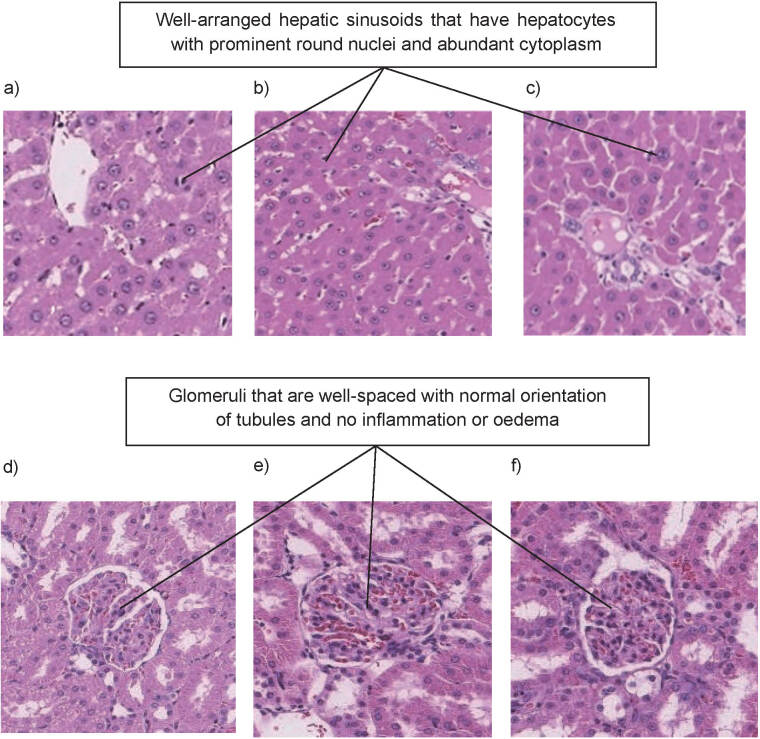
Photomicrographs of liver cross-sections of: a) normal control, b) diabetic control (b), c) rats treated with supercoagulant curd, and kidney cross-sections of: d) normal control, e) diabetic control and f) rats treated with supercoagulant curd at 10× magnification

The liver of all rats from the NC, DC and SC groups, as shown in Figs. 5a, 5b and 5c, respectively, had normal morphology and no signs of damage. The central vein was prominent, and the hepatic sinusoids were well arranged amidst healthy parenchyma, which consisted of hepatocytes with prominent round nuclei and abundant cytoplasm. Untreated diabetes is usually associated with damage to the liver, as it increases oxidative stress and causes an aberrant inflammatory response that activates the transcription of proapoptotic genes and damages hepatocytes in the liver (*42*). It was found that the DC group had no visible overall structural changes as evaluated by the pathologist. These observations are common in an acute study. The duration of the current study was only 20 days and hence generalized as an acute rather than a chronic study, which is usually 8 to 10 weeks (*43*, *44*). Even though the liver function test revealed a preliminary elevation of biochemical markers in the blood, an acute study period of 20 days is insufficient to cause observable liver damage. According to a previous study, enlargement of the sinusoids and focal microvesicular fatty degeneration with vacuolization of liver cells could be seen only in the 6th week of diabetes (*45*).

Diabetes causes an accumulation of glucose in the body, and over time, a high level of glucose will damage the blood vessel clusters called glomeruli in the kidney, which filters waste from the blood (*46*). The kidneys of the rats in NC, DC and SC groups (Figs. 5d, 5e and 5f, respectively) showed adequate glomeruli that were well spaced with normal orientation of the tubules. The interstitium had no inflammation or oedema, and the tubules were empty without casts or haemorrhage. The glucose in the blood of the DC group did not cause observable damage to the kidneys 20 days after induction of diabetes; however, the renal function test revealed a preliminary elevation of biochemical markers of the urea and serum creatinine levels in the blood. The SC group showed similar normal kidney morphology without any observable damage. Such an outcome is attributed to the ability of the used plants to maintain low blood glucose level. Generally, most medicinal plants have been indicated to be protective towards vital organs such as liver (*47*), however, this should be evaluated over a longer period, *i.e.* in a chronic toxicological study. In histopathological evaluation of liver and kidney in this study, the curd obtained with noni, pineapple and mango as coagulants had no immediate toxic effect on liver and kidneys.

## CONCLUSIONS

This study focused on finding a plant-based coagulant with dual effects of coagulating milk to form curd and simultaneously giving the curd medicinal properties. *Morinda citrifolia* (noni), *Ananas comosus* (pineapple) and *Mangifera indica* (mango) were mixed to form a supercoagulant. The supercoagulant demonstrated good milk coagulation ability, which was evident in comparison with rennet. Supercoagulant-fortified curd was also found to have significant *in vitro* and *in vivo* biological activities in rats. Histopathological study was also performed on the liver and kidney of the rats, and no immediate damage was observable, which indicates that the curd is safe for consumption. From the results, it can be concluded that these supercoagulants can be used as a raw material for preparing functional dairy food with dual effects of coagulating milk and antidiabetic activity. The obtained products could be used to supplement or replace dairy products with the aim to prevent diabetes or control the blood sugar level in diabetic patients.

## References

[1] Dalgleish DG. On the structural models of bovine casein micelles—Review and possible improvements. Soft Matter. 2011;7(6):2265–72. 10.1039/C0SM00806K

[2] Shah MA Mir SA Paray MA. Plant proteases as milk-clotting enzymes in cheesemaking: A review. Dairy Sci Technol. 2014;94(1):5–16. 10.1007/s13594-013-0144-3

[3] Bathmanathan R Yahya Y Yusoff M Vejayan J. Utilizing coagulant plants in the development of functional dairy foods and beverages: A mini review. J Biol Sci. 2019;19(3):259–71. 10.3923/jbs.2019.259.271

[4] Diabetes facts & figures. Brussels, Belgium: International Diabetes Federation; 2019. Available from: https://idf.org/aboutdiabetes/what-is-diabetes/facts-figures.html.

[5] Poovitha S, Parani M. In vitro and in vivo α-amylase and α-glucosidase inhibiting activities of the protein extracts from two varieties of bitter gourd (*Momordica charantia* L.). BMC Complement Med Ther. 2016;16(1):185. https://doi.org/10.1186/s12906-016-1085-1 10.1186/s12906-016-1085-1 PMC4959359 27454418

[6] Bahadoran Z Tohidi M Nazeri P Mehran M Azizi F Mirmiran P. Effect of broccoli sprouts on insulin resistance in type 2 diabetic patients: A randomized double-blind clinical trial. Int J Food Sci Nutr. 2012;63(7):767–71. 10.3109/09637486.2012.665043 22537070

[7] Crawford P. Effectiveness of cinnamon for lowering hemoglobin A1C in patients with type 2 diabetes: A randomized, controlled trial. J Am Board Fam Med. 2009;22(5):507–12. 10.3122/jabfm.2009.05.080093 19734396

[8] Nabavi SF Thiagarajan R Rastrelli L Daglia M Sobarzo-Sanchez E Alinezhad H Curcumin: A natural product for diabetes and its complications. Curr Top Med Chem. 2015;15(23):2445–55. 10.2174/1568026615666150619142519 26088351

[9] Lucey JA Fox P. Importance of calcium and phosphate in cheese manufacture: A review. J Dairy Sci. 1993;76(6):1714–24. 10.3168/jds.S0022-0302(93)77504-9

[10] Yilmazer-Musa M Griffith AM Michels AJ Schneider E Frei B. Grape seed and tea extracts and catechin 3-gallates are potent inhibitors of α-amylase and α-glucosidase activity. J Agric Food Chem. 2012;60(36):8924–9. 10.1021/jf301147n 22697360 PMC4356113

[11] Ademiluyi AO Oboh G. Soybean phenolic-rich extracts inhibit key-enzymes linked to type 2 diabetes (α-amylase and α-glucosidase) and hypertension (angiotensin I converting enzyme) *in vitro.* Exp Toxicol Pathol. 2013;65(3):305–9. 10.1016/j.etp.2011.09.005 22005499

[12] Di Filippo C Marfella R Cuzzocrea S Piegari E Petronella P Giugliano D Hyperglycemia in streptozotocin-induced diabetic rat increases infarct size associated with low levels of myocardial HO-1 during ischemia/reperfusion. Diabetes. 2005;54(3):803–10. 10.2337/diabetes.54.3.803 15734859

[13] Vejayan J Yahya YAC Chakravarthi S Bathmanathan R Ibrahim H Yun A. Tongkat ali plants of *Eurycoma longifolia* and *Stema tuberosa* stimulate sexual arousal in domestic cocks. Malays J Sci. 2020;39(1):1–14. 10.22452/mjs.vol39no1.1

[14] IBM SPSS Statistics for Windows, v. 25.0, IBM Corp., Armonk, NY, USA; 2017.

[15] de Farias VA da Rocha Lima AD Costa AS de Freitas CDT da Silva Araújo IM dos Santos Garruti D Noni (*Morinda citrifolia* L.) fruit as a new source of milk-clotting cysteine proteases. Food Res Int. 2020;127:108689. 10.1016/j.foodres.2019.108689 31882081

[16] Komansilan S Rosyidi D Radiati LE Purwadi P Evanuarini H. The physicochemical characteristics and protein profile of cottage cheese produced by using crude bromelain enzyme extracted from *Ananas comosus.* Curr Res Nutr Food Sci. 2021;9(2):578–87. 10.12944/CRNFSJ.9.2.21

[17] Vejayan J Munir N Lian YL Bathmanathan R Ibrahim H Chakravarthi S. Tropical fruits of mango and noni having dual effects of coagulating milk and enriching the curds with micro-constituents of medicinal potential. Malays J Sci. 2019;38(3):34–7. 10.22452/mjs.vol38no3.4

[18] Hallén E Lundén A Allmere T Andrén A. Casein retention in curd and loss of casein into whey at chymosin-induced coagulation of milk. J Dairy Res. 2010;77(1):71–6. 10.1017/S0022029909990434 19939322

[19] McMahon DJ Brown R Ernstrom CA. Enzymic coagulation of milk casein micelles. J Dairy Sci. 1984;67(4):745–8. 10.3168/jds.S0022-0302(84)81364-8

[20] Vejayan J Zulkifli AA Syed M Saufi NM Ibrahim H Ambu S. Uncovering a protease in snake venom capable to coagulate milk to curd. Int J Adv Biotechnol Res. 2017;8(4):409–23.

[21] Amira AB Besbes S Attia H Blecker C. Milk-clotting properties of plant rennets and their enzymatic, rheological, and sensory role in cheese making: A review. Int J Food Prop. 2017;20(1):S76–93. 10.1080/10942912.2017.1289959

[22] Soltani M Boran O Hayaloglu A. Effect of various blends of camel chymosin and microbial rennet (*Rhizomucor miehei*) on microstructure and rheological properties of Iranian UF white cheese. Lebensm Wiss Technol. 2016;68:724–8. 10.1016/j.lwt.2016.01.028

[23] Sandra S Ho M Alexander M Corredig M. Effect of soluble calcium on the renneting properties of casein micelles as measured by rheology and diffusing wave spectroscopy. J Dairy Sci. 2012;95(1):75–82. 10.3168/jds.2011-4713 22192185

[24] McMahon DJ, Oommen BS. Casein micelle structure, functions, and interactions. In: McSweeney P, Fox P, editors. Advanced dairy chemistry. Boston, MA, USA: Springer; 2013. pp. 185-209. https://doi.org/10.1007/978-1-4614-4714-6_6 10.1007/978-1-4614-4714-6_6

[25] Simpson BK, editor. Food biochemistry and food processing. Hoboken, NJ, USA: John Wiley & Sons; 2012. https://doi.org/10.1002/9781118308035 10.1002/9781118308035

[26] Khan N Choi JY Nho EY Hwang IM Habte G Khan MA Determination of mineral elements in milk products by inductively coupled plasma-optical emission spectrometry. Anal Lett. 2014;47(9):1606–13. 10.1080/00032719.2013.878842

[27] Irondi EA Oboh G Akindahunsi AA Boligon AA Athayde ML. Phenolic composition and inhibitory activity of *Mangifera indica* and *Mucuna urens* seeds extracts against key enzymes linked to the pathology and complications of type 2 diabetes. Asian Pac J Trop Med. 2014;4:903–10. 10.12980/APJTB.4.201414B364

[28] Westendorf J Mettlich C. The benefits of noni juice: An epidemiological evaluation in Europe. J Med Food Plants. 2009;1(2):64–79. 10.3390/foods7040058

[29] Horsfall AU Olabiyi O Aiyegbusi A Noronha C Okanlawon A. *Morinda citrifolia* fruit juice augments insulin action in sprague–dawley rats with experimentally induced diabetes. Nig Q J Hosp Med. 2008;18:162–5. 19062482 10.4314/nqjhm.v18i3.45020

[30] Chuah AM Lee YC Yamaguchi T Takamura H Yin LJ Matoba T. Effect of cooking on the antioxidant properties of coloured peppers. Food Chem. 2008;111(1):20–8. 10.1016/j.foodchem.2008.03.022

[31] Isabelle M Lee B Lim M Koh WP Huang D Ong C. Antioxidant activity and profiles of common fruits in Singapore. Food Chem. 2010;123:77–84. 10.1016/j.foodchem.2010.04.002

[32] Lonergan SM, Topel DG, Marple DN, editors. The science of animal growth and meat technology. Cambridge, MA, USA: Academic Press; 2018. https://doi.org/10.1016/C2017-0-02648-4 10.1016/C2017-0-02648-4

[33] Pournaghi P Sadrkhanlou RA Hasanzadeh S Foroughi A. An investigation on body weights, blood glucose levels and pituitary-gonadal axis hormones in diabetic and metformin-treated diabetic female rats. Vet Res Forum. 2012;3(2):79–84. 25653751 PMC4312800

[34] Seedevi P Ganesan AR Moovendhan M Mohan K Sivasankar P Loganathan S Anti-diabetic activity of crude polysaccharide and rhamnose-enriched polysaccharide from *G. lithophila* on streptozotocin (STZ)-induced in Wistar rats. Sci Rep. 2020;10(1):556. 10.1038/s41598-020-57486-w 31953455 PMC6969100

[35] Nair SS Kavrekar V Mishra A. *In vitro* studies on α-amylase and α-glucosidase inhibitory activities of selected plant extracts. Eur J Exp Biol. 2013;3(1):128–32.

[36] Hasan KMM Tamanna N Haque MA. Biochemical and histopathological profiling of Wistar rat treated with *Brassica napus* as a supplementary feed. Food Sci Hum Wellness. 2018;7(1):77–82. 10.1016/j.fshw.2017.12.002

[37] Giannini EG Testa R Savarino V. Liver enzyme alteration: A guide for clinicians. CMAJ. 2005;172(3):367–79. 10.1503/cmaj.1040752 15684121 PMC545762

[38] Silverstein D, Hopper K, editors. Small animal critical care medicine. Amsterdam, The Netherlands: Elsevier Inc; 2008.

[39] Park KT Yun CH Bae CS Ahn T. Decreased level of albumin in peripheral blood mononuclear cells of streptozotocin-induced diabetic rats. J Vet Med Sci. 2014;76(8):1087–92. 10.1292/jvms.13-0631 24758836 PMC4155187

[40] Ayeleso A Brooks N Oguntibeju O. Modulation of antioxidant status in streptozotocin-induced diabetic male Wistar rats following intake of red palm oil and/or rooibos. Asian Pac J Trop Med. 2014;7(7):536–44. 10.1016/S1995-7645(14)60090-0 25063283

[41] Kazeem MI Akanji MA Yakubu MT. Amelioration of pancreatic and renal derangements in streptozotocin-induced diabetic rats by polyphenol extracts of ginger (*Zingiber officinale*) rhizome. Pathophysiology. 2015;22(4):203–9. 10.1016/j.pathophys.2015.08.004 26349770

[42] Mohamed J Nafizah AN Zariyantey A Budin S. Mechanisms of diabetes-induced liver damage: The role of oxidative stress and inflammation. Sultan Qaboos Univ Med J. 2016;16(2):e132–41. 10.18295/squmj.2016.16.02.002 27226903 PMC4868511

[43] Pourghasem M Nasiri E Shafi H. Early renal histological changes in alloxan-induced diabetic rats. Int J Mol Cell Med. 2014;3(1):11–5. 24551816 PMC3927393

[44] Volper BD Huynh RT Arthur KA Noone J Gordon BD Zacherle EW Influence of acute and chronic streptozotocin-induced diabetes on the rat tendon extracellular matrix and mechanical properties. Am J Physiol Regul Integr Comp Physiol. 2015;309(9):R1135–43. 10.1152/ajpregu.00189.2015 26310937

[45] Bilal HM Riaz F Munir K Saqib A Sarwar MR. Histological changes in the liver of diabetic rats: A review of pathogenesis of nonalcoholic fatty liver disease in type 1 diabetes mellitus. Cogent Med. 2017;3(1):1275415. 10.1080/2331205X.2016.1275415

[46] Gross JL De Azevedo MJ Silveiro SP Canani LH Caramori ML Zelmanovitz T. Diabetic nephropathy: Diagnosis, prevention, and treatment. Diabetes Care. 2005;28(1):164–76. 10.2337/diacare.28.1.164 15616252

[47] Abou Seif HS. Physiological changes due to hepatotoxicity and the protective role of some medicinal plants. Beni Suef Univ J Basic Appl Sci. 2016;5(2):134–46. 10.1016/j.bjbas.2016.03.004

