# Corrigendum to: Fruit Extract Derived from a Mixture of Noni, Pineapple and Mango Capable of Coagulating Milk and Producing Curd with Antidiabetic Activities (Published: Food Technol. Biotechnol. 60 (3) 375-385 (2022) https://doi.org/10.17113/ftb.60.03.22.7456)

**DOI:** 10.17113/ftb.60.03.22.7456_corrig.61(3)

**Published:** 2023-09

**Authors:** Jaya Vejayan, Rupbansraaj Bathmanathan, Sharifah Aminah Tuan Said, Srikumar Chakravarthi, Halijah Ibrahim

**Affiliations:** 1 Faculty of Industrial Sciences & Technology, Universiti Malaysia Pahang, Lebuhraya Tun Razak, 26300, Gambang, Kuantan, Pahang Darul Makmur, Malaysia; 2 Faculty of Medicine, Biomedical Sciences and Nursing, MAHSA University, Jalan SP2, Bandar Saujana Putra, 42610 Jenjarom, Selangor, Malaysia; 3 Institute of Biological Sciences, University of Malaya, 50603 Kuala Lumpur, Malaysia

The authors request that the Funding section be amended to conform to the correct format required by the funding institution, *i.e.* the Ministry of Higher Education of Malaysia.

Previously written statement in the funding section:

Funding for this work was provided by Ministry of Education, Malaysia, with Fundamental Research Grant Scheme (FRGS) with external reference number FRGS/1/2022/STG01/ UMP/02/1 and title: Investigations of Protein from the Lesser Known Tongkat Ali Plants of *Stema tuberosa* and *Polyalthia bullata* for Their Potentials in Improving Men’s Health. The present study was the outcome of using chemicals and consumables purchased from this grant without compromising its main objectives and milestones.

is changed to:

The authors would like to thank Ministry of Higher Education for providing financial support under Fundamental Research Grant Scheme (FRGS) No: FRGS/1/2022/STG01/UMP/02/1 (University reference RDU220110) and Universiti Malaysia Pahang for laboratory facilities. The title of the FRGS grant: Investigations of Protein from the Lesser Known Tongkat Ali Plants of *Stema tuberosa* and *Polyalthia bullata* for Their Potentials in Improving Men’s Health. The present study was the outcome of using chemicals and consumables purchased from this grant without compromising its main objectives and milestones.

